# Percutaneous Left Atrial Appendage Occlusion Therapy: Evolution and Growing Evidence

**DOI:** 10.31083/j.rcm2407211

**Published:** 2023-07-19

**Authors:** Xinqiang Han, David G. Benditt

**Affiliations:** ^1^Cardiology Division of Reid Health, Indiana University School of Medicine, Richmond, IN 47374, USA; ^2^Cardiac Arrhythmia Center, Cardiovascular Division, Department of Medicine, University of Minnesota Medical School, Minneapolis, MN 55455, USA

**Keywords:** atrial fibrillation, left atrial appendage occlusion, stroke prevention, oral anticoagulation

## Abstract

Atrial fibrillation (AF) is the most common cardiac arrhythmia and if untreated, significantly increases both the risk of intracardiac thrombus formation and ischemic stroke. In patients with nonvalvular AF (NVAF), the left atrial appendage (LAA) has been estimated to be the source of thrombus development in 91% to 99% of cases. Consequently, oral anticoagulation (OAC) to provide stroke prevention has become the standard of care for most AF patients; however, OACs are associated with a risk of bleeding and their efficacy depends on optimal patient compliance. In terms of alternative approaches to preventing embolic events, surgical LAA excision was attempted as early as in the late 1940s in patients with valvular AF; LAA excision remains a recommendation in surgical guidelines for NVAF patients who need open-heart coronary bypass or valvular replacement/repair surgeries. However, due to its invasive nature surgical LAA intervention has limited clinical application in present cardiology practice. Percutaneous LAA occlusion (LAAO) is increasingly being performed as an alternative to OAC for stroke prevention; this is particularly the case in patients at increased bleeding risk. Substantial progress has been made in percutaneous LAAO therapy since its inception some twenty years ago. Herein we systematically review both the critical literature that led to the development of LAAO, and the increasing clinical evidence supporting the application of this treatment strategy in NVAF. To this end we focus on recently published critical evaluations of United States Food and Drug Administration (US FDA) and Conformité Européenne (Commercial Sale of Licensed Product in the EU) (CE-Mark) approved LAAO devices, summarize the current status of LAAO therapy, and discuss the future perspectives regarding the knowledge and technology gaps in this area by recognizing the potential contributions of many ongoing but likely transformative clinical trials.

## 1. Introduction

Thromboembolic complications, particularly stroke, are among the most important adverse events associated with atrial fibrillation (AF) [1, 2, 3], and the left atrial appendage (LAA), with its muscular trabeculations and often complex multilobular structure has long been considered the principal site of atrial clot formation [4, 5]. Consequently, apart from pharmacologic prevention of thrombus formation and embolization being a standard of care consideration in the long-term treatment of most individuals with AF [6, 7], there also exists a substantial body of clinical experience addressing the other methods of diminishing LAA-induced embolic risk, including device therapy [8]. In this context, a degree of thrombotic risk reduction has been achieved by techniques that modify the LAA anatomy to reduce its capacity to facilitate thrombus formation. These techniques began with surgical methods to amputate the LAA, or by suturing and closing the LAA ostium [9, 10] with the objective of eliminating a clot provoking LAA from releasing thrombi into the central systemic circulation. While a degree of success has been reported by these surgical approaches as discussed below, their utility is limited by their invasive nature. Later, more readily applicable catheter based LAA occlusion (LAAO) systems were introduced and have gradually gained importance.

The goal of this review is to examine the role that the LAA may play in intra-atrial thrombus development in AF and summarize the recent evolution of the LAAO therapy. Emphasis is focused on the increasing evidence favoring trans-catheter LAAO given its potential value for stroke prevention in many AF patients who cannot tolerate or have contraindications to long-term conventional oral anticoagulation.

## 2. Pertinent Terminology and Anatomy

### 2.1 Nonvalvular AF

The term nonvalvular AF (NVAF), sometimes also called nonrheumatic AF, has been used since the 1970s to differentiate AF in association with rheumatic heart disease from AF in the absence of rheumatic heart disease. The European Society of Cardiology (ESC) defined it as AF in the absence of “rheumatic native or prosthetic heart valves” in 2012. Shortly afterward the American Heart Association (AHA)/American College of Cardiology (ACC)/ Heart Rhythm Society (HRS) 2014 guideline refined the definition to AF occurring in the absence of “rheumatic mitral stenosis, mitral valve repair, mechanical, or bioprosthetic heart valve”. The current definition of “valvular AF” only applies to AF in the presence of any mechanical heart valve or AF in the presence of moderate to severe mitral stenosis, rheumatic or nonrheumatic in etiology. The current definition is accepted by AHA/ACC/HRS and ESC, but the ESC goes further to recommend that the term NVAF be abandoned. As a result, it is evident that AF associated with severe mitral regurgitation or aortic stenosis is not included in the “valvular” AF by the current definition, unless a mechanical valve has been placed in the patient for whatever etiology. For historic description both “valvular” and “rheumatic” will be used in this review to differentiate the subtype of AF from “nonvalvular” AF.

### 2.2 Left Atrial Appendage

The LAA is generally regarded as a vestigial remnant of the primordial left atrium which forms during the fourth week of embryonic development. Detailed discussions of LAA anatomy, physiology, and pathophysiology can be found in excellent reviews [11, 12, 13]. In general, the hook-like diverticulum of LAA consists of one or more lobes with a trabeculated wall due to parallel-running pectinate muscles [14, 15]. In health, the LAA is a highly contractile structure (contracts from its apex toward the base) and in sinus rhythm the blood flow within the LAA lumen is sufficient to minimize thrombus formation. However, during AF, the contractility of the LAA is markedly reduced and the blood flow within the lumen may become sufficiently slow favoring thrombus formation [16, 17]. The highly trabeculated wall of the LAA, and the often-concomitant presence of fibrous tissue in the LAA and atria in AF patients also likely play an important part in thrombogenicity. As such, the fibrillating trabeculated LAA with stasis of blood facilitates coagulation activation and elevates the risk of thromboembolism leading to an overall risk of stroke of approximately 5% every year [1, 3].

In the 1950s when rheumatic valve disease was the main cause of AF, it became recognized that the LAA was responsible for about 50% of thromboses, with a consequent 50% embolic risk reduction after LAA obliteration at the time of the commissurotomy [18]. By the mid 1990’s with the extensive clinical application of transesophageal echocardiography (TEE), analyses suggested that left atrial (LA) thrombi were present in LA cavity or were present in the LAA and extended into the cavity in 57% of patients with rheumatic AF. However, in nonrheumatic AF, about 90% of thrombi were largely isolated to the LAA [4]. As for NVAF, it had become clear by the late 1970s that increased risk of ischemic stroke was associated with AF in the absence of significant valvular heart diseases [19, 20, 21]. The most recent data suggests that about 99% of thrombi in NVAF are formed in the LAA [5].

The first amputations of the LAA in humans [22] were reported shortly after the procedure was performed in animal experiments in the late 1940’s [23, 24]. After these successful pioneering attempts, the procedure was subsequently performed at the time of mitral commissurotomy, to alleviate the well-known high thrombogenicity associated with mitral stenosis [18, 25]. In facts concomitant surgical excision of the LAA has been recently recommended in addition to ablation procedures in surgical guidelines [26].

Currently the LAA exclusion procedure is commonly performed by resection, epicardial stapling, clip application, or endo-atrial double-layer longitudinal suture closure at the time of open-heart surgery for coronary bypass or valvular repair/replacement [27, 28, 29]. Stapling appears to have particularly poor outcomes, with many patients having a residual LAA stump and/or surgical line leakage, which can be thrombogenic. LAA obliteration may reduce early and late stroke rates by more than 50% and have modest survival benefit [10]. The potential thrombogenicity of the remnant appendage pouch is a matter of major concern irrespective of the surgical methods for LAA exclusion [30, 31, 32]. In a nonrandomized retrospective study that compared the efficacy of several surgical methods of LAA closure, TEE revealed a successful closure in only 40% of the patients [33]. LAA thrombus was present in 41% with unsuccessful LAA exclusion. Importantly, 13% of these patients had suffered strokes in the time from the operation to when TEE was performed, be it successful or unsuccessful closure [33]. Despite these shortcomings and less than ideal outcomes, the recent LAAO III trial further supports the efficacy of surgical LAA obliteration in ischemic stroke prevention in NVAF patients [34].

## 3. LAAO Devices

The stimulus for investigating the possibility of percutaneous LAA obliteration or occlusion was fourfold: (1) As noted earlier, thrombus associated with nonrheumatic AF occurs predominantly within the LAA in 91–99% of patients [4, 5]. (2) There are many patients in whom anticoagulant drugs (warfarin or novel oral anticoagulants/direct oral anticoagulants [NOACs/DOACs]) are not suitable as therapy to reduce embolic stroke because of relative or absolute contraindications, particularly bleeding disorders. Additionally, real-world experience indicates that adherence to anticoagulation is far from optimal, thereby leaving many patients unprotected [35, 36]. (3) Even in patients with chronic anticoagulation using either warfarin or NOACs/DOACs there remains substantial risk of thrombus formation in LAA despite medication compliance [34, 37, 38, 39, 40]. (4) Surgical approaches are more invasive making their widespread application inappropriate for most AF patients, apart from the residual remaining risks associated with remnants of the LAA or residual leakage regardless of the surgical exclusion methods. In the following discussion the major LAAO devices will be described in chronological order. Timelines of device preclinical, United States Food and Drug Administration (US FDA) and Conformité Européenne (Commercial Sale of Licensed Product in the EU) (CE-Mark) approval, and main relevant studies are summarized in Table 1 (Ref. [41, 42, 43, 44, 45, 46, 47, 48, 49, 50, 51, 52, 53, 54, 55, 56, 57, 58, 59, 60, 61, 62, 63, 64, 65, 66, 67, 68, 69, 70, 71, 72, 73, 74, 75, 76, 77, 78, 79, 80, 81, 82, 83, 84, 85, 86, 87, 88, 89, 90, 91, 92, 93, 94, 95, 96, 97, 98, 99, 100]).

**Table 1.** **LAAO devices in clinical use or trials with main studies referenced**.

Devices		Preclinical	Studies	FDA	CE-Mark	Withdrawn
PLAATO		2001	Refs. [41, 42, 43, 44, 45, 46, 47, 48]			2007
Amplatzer						
	Occluder	2002	Ref. [49]			
	ACP I	2008	Refs. [50, 51, 52, 53, 54, 55]		2008	
	Amulet	2012	Refs. [52, 53, 54, 55, 56, 57, 58]	2020	2013	
Watchman						
	2.5	2005	Refs. [59, 60, 61, 62, 63, 64, 65, 66, 67, 68, 69, 70, 71]	2015	2005	2021
	FLX	2015	Refs. [72, 73]	2020	2015	
LAmbre		2013	Refs. [74, 75, 76, 77, 78, 79]		2016	
WaveCrest		2010–2011	Refs. [80, 81]		2013	
LARIAT		2010	Refs. [82, 83, 84, 85, 86, 87, 88, 89, 90, 91, 92]	2006	2015	
				2009		
				2014		
Ultraseal I/II		2015–2016	Refs. [93, 94, 95, 96, 97]		2016	
CLAAS		2021	Refs. [98, 99, 100]			

Abbreviations: FDA, food and drug administration (US); CE-Mark, Conformité Européenne (Commercial Sale of Licensed Product in the EU); PLAATO, percutaneous left atrial appendage transcatheter occlusion; ACP, Amplatzer cardiac plug; CLAAS, Conformal left atrial appendage seal; LAAO, left atrial appendage occlusion.

### 3.1 PLATTO: Early Stage Percutaneous LAAO

Following pilot feasibility study in animals [41], the first percutaneous left atrial appendage transcatheter occlusion device in human was described two decades ago [42] with the detailed technique for implantation being summarized a decade ago [43]. The device was made of a self-expanding nitinol cage covered with an expanded polytetrafluoroethylene (ePTFE) membrane. The implant was available with diameters of 15 to 32 mm and delivered through a 12 F transseptal sheath under a combination of TEE guidance and fluoroscopy. With this approach, LAA was successfully occluded in 15 out of 15 “chronic” AF patients having a contraindication to warfarin (average age 69 ± 5 years). TEE and chest X-ray confirmed stable implant position with smooth atrial-facing surface and no evidence of thrombus at one month follow-up. At 6-month follow-up, percutaneous LAA occlusion (PLAATO) continued to achieve an adequate seal of the neck of the LAA without apparent effect on the structure or function of the atrium and left upper pulmonary vein.

A prospective, non-randomized, multi-center trial of PLAATO enrolling 111 patients from August 2001 to November 2003 was published in 2005 [44]. With an average follow-up of 9.8 months, the study demonstrated an overall procedure success in 108 out of 111 patients (97.3%) with no migration or mobile thrombus on TEE at one and six months after device implantation. Three patients in the study did not receive a PLAATO device: one with left atrial thrombus at the time of the procedure, one because of vessel perforation during venous access, and a third who developed pericardial effusion causing tamponade after trans-septal puncture. The conclusion was that the percutaneous LAAO using the PLAATO system could be performed at acceptable risk, and that this approach provided an alternative therapeutic option for patients with AF who were at increased risk for ischemic stroke but who had a contraindication to long-term warfarin treatment. Additional studies in small and medium numbers of high stroke risk AF patients reinforced the concept that LAAO using PLAATO was relatively safe and effective although severe complications could occur [45, 46, 47, 48]. Despite its apparent effectiveness, the PLAATO device has not been available since 2007; its absence has been due to commercial and not medical reasons.

### 3.2 Amplatzer™ Septal Occluder, Cardiac Plug, and Amulet

**Fig. 1.**
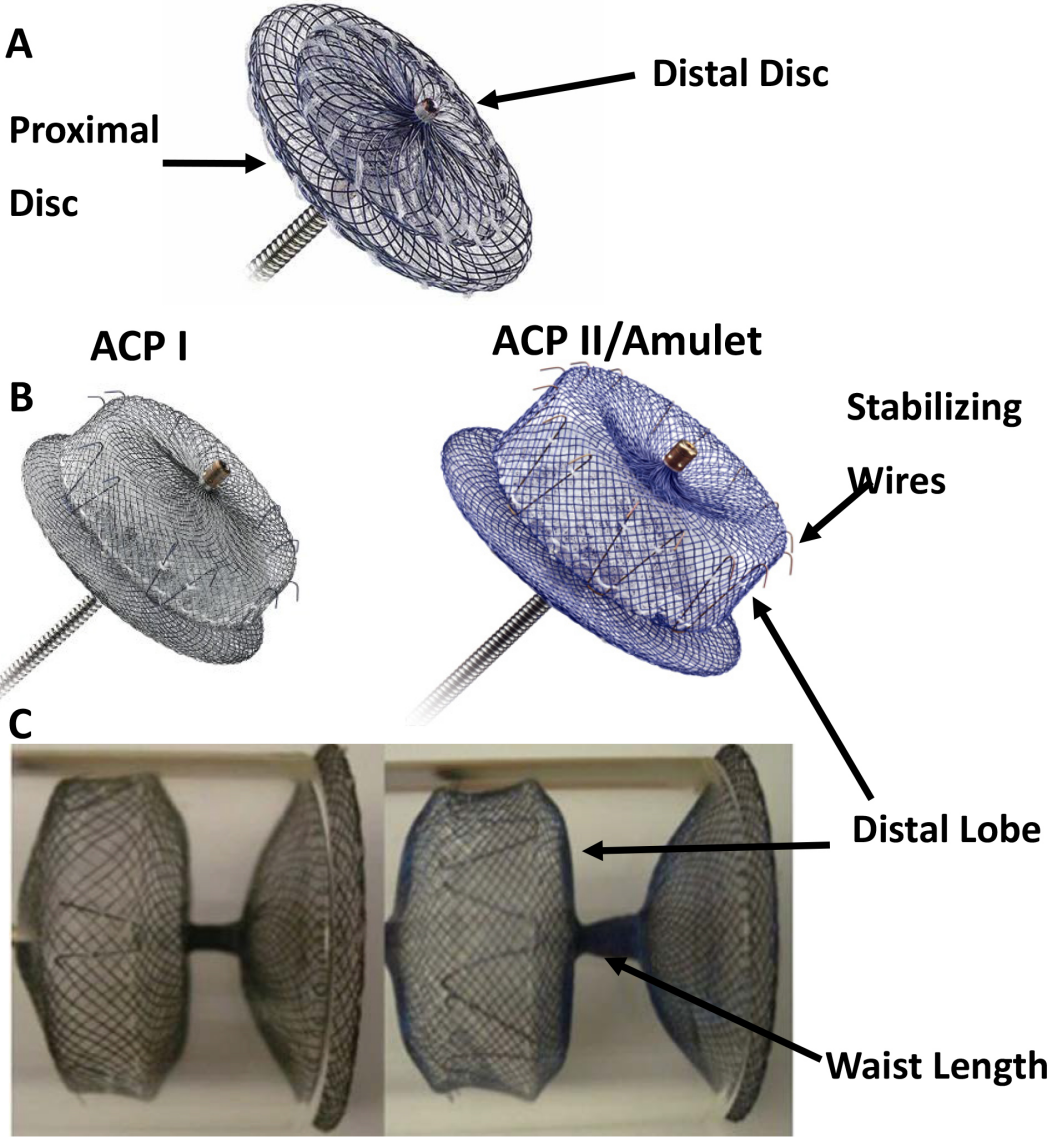
**Amplatzer Septal Occluder (A), ACP I, and ACP II (Amulet) devices (B,C)**. Key feature is the double-disc design. Major differences between ACP I and ACP II include: for the later (1) the stabilizing hooks are stiffer and increased from six pairs to up to 10 pairs; (2) the length of the distal lobe and the diameter of the proximal disc have been increased; (3) the waist between the distal lobe and the proximal disc has been lengthened; and (4) the attaching screw on the proximal disc has been inverted (From St Jude Medical). ACP, Amplatzer cardiac plug.

The first study of LAAO with Amplatzer atrial septal occlusion devices (Fig. 1A) was published in 2003 [49]. A total of 16 patients with NVAF aged 58 to 83 years were treated at four centers, with 14 of the patients receiving only local anesthesia. One developed acute device embolization requiring surgical removal. At 4 months follow-up, there were no further complications; the devices remained in stable position and the LAA was completely occluded in all cases. It should be noted that the device was initially developed for atrial septal defect closure and not specifically designed for LAAO purpose. There were no further clinical data using the septal occlusion device until a subsequent system, the Amplatzer Cardiac Plug (ACP I) was specifically designed for occlusion of the LAA [50, 51, 52]. The device (Fig. 1B,C, left) was made from a nitinol mesh and Dacron in a lob and disc design, with 12 stabilizing wires equally spaced about the main disc. The sizes of the lobes ranged between 16 to 30 mm. This device was retrievable and could be repositioned with successful deployment confirmed by intraprocedural TEE [51].

Most of the clinical data for ACP I came from the ACP multicenter registry [53], with findings obtained in 1047 consecutive patients from 22 centers between December 2008 and November 2013. A total of 1001 patients who underwent LAAO with the ACP I device had complete follow-up. Clinical outcomes including stroke rate and bleeding reduction in the device patients were analyzed by comparing with their predicted risks by the CHA2DS2-Vasc (Congestive heart failure, Hypertension, Age 65/75, Diabetes, Stroke/transient ischemic attack (TIA), Coronary artery disease or peripheral arterial disease) and HAS-BLED (Hypertension, Abnormal liver or kidney function, Stroke/TIA, Bleeding tendency or prior major bleeding, labile international normalized ratio (INR), Elderly ≥65 years, Drugs/Alcohol) scores, respectively. Mean follow-up was 13 months. Procedural success was achieved in 1019/1047 patients (97.3%) with a total of 52 peri-procedural major adverse events (4.97%) including deaths, strokes/transient ischemic attacks (TIAs), and cardiac tamponades. The study findings must be considered in light of a number of limitations, including: (1) non-randomized design (no control group); (2) incomplete TEE follow-up; and (3) self-reporting results without independent verification.

First generation ACP I major complications [53, 54, 55] included peri-procedural stroke (0 to 2.3%), device embolization (0 to 2.3%), device thrombosis (0 to 2.4%), and pericardial effusion (1.1 to 3.5%). These adverse events mandated that further technological improvements be made. Consequently, a new generation device from Amplatzer, Amulet (or ACP II) has been designed (Fig. 1B,C, right), without changing the main frame of the ACP I. The modifications were made to facilitate device implantation and improve device sealing of the appendage after implantation. The first in-human percutaneous LAAO using Amulet was performed in 2012 and published one year later [52]. A multicenter prospective real-world registry study including 1088 patients with NVAF was published in 2017 [56]. In this latter population, long-term anticoagulation was contraindicated in 82.8% and previous major bleeding occurred in 72.4%. Device implantation was successfully achieved in 99.0% and major adverse events including death, major bleeding, tamponade requiring pericardial drainage or surgery, significant vascular complications, stroke, and device embolization occurred in 3.2% of patients during the index hospitalization. Available TEE follow-up in 673 patients post-implantation showed adequate (<3 mm jet) occlusion of the appendage in 98.2% and device thrombus in 1.5%. Potential selection bias and the fact that only approximately 62% of the study population had follow-up TEE may have been important limitations. Nonetheless, in this study population a total of 1078 patients did successfully receive an Amulet device. When compared to a propensity score-matched control cohort of 1184 NVAF treated by direct oral anticoagulants (NOACs /DOACs), at 2-year follow-up LAAO with Amulet was found to have similar stroke prevention efficacy, but lower risk of major bleeding and mortality after analyzing the primary outcome composite of ischemic stroke, major bleeding, or all-cause mortality [57]. After the investigational device exemption (IDE) trial [58] confirming the noninferiority for safety and effectiveness of stroke prevention comparing with the first US FDA approved Watchman™ Legacy (March 2015) Amulet was approved by US FDA in August 2020. Procedure-related complications were noticed to be higher with the Amulet occluder in earlier implants and decreased with operator experience.

### 3.3 Watchman 2.5/Legacy

**Fig. 2.**
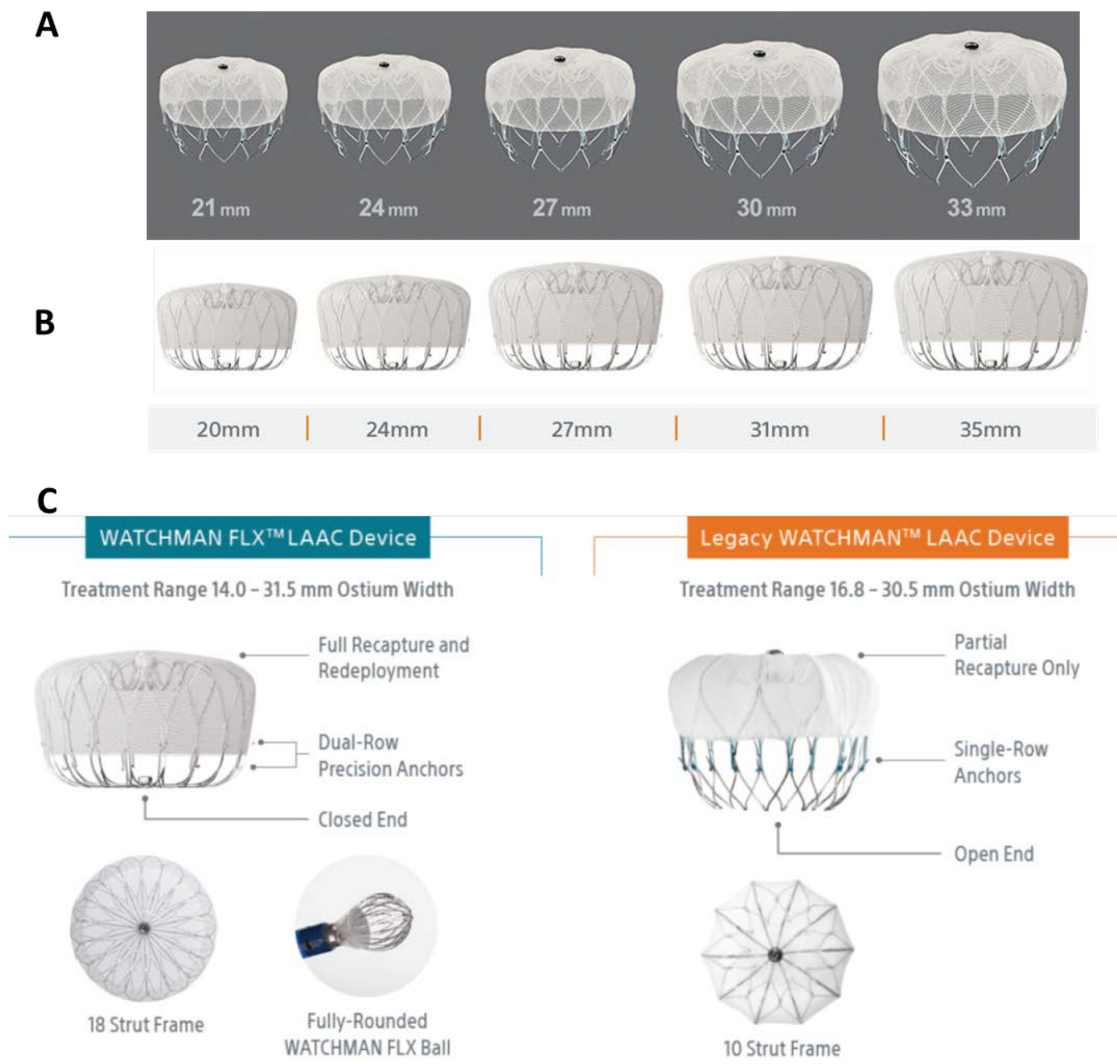
**The 1st and the 2nd generation Watchman devices**. (A) Watchman 2.5 (Legacy). (B) Watchman FLX. (C) Comparison of the detailed parameters. Watchman 2.5 has been off the US market since the first quarter of 2021 (From Boston Scientific). LAAC, left atrial appendage closure.

Description of the Watchman device (Watchman 2.5 or Legacy) was first published in 2006 [59, 60]. Enrollment of PROTECT AF trial started in February 2005 and ended in the summer of 2008. The pilot data was published in 2007 [61] and the complete study in 2009 [62]. The Watchman left atrial appendage system includes an implant/device, a delivery sheath (14 F), and a catheter (12 F). Watchman implant consists of a self-expanding nitinol frame and a permeable polyester fabric (Fig. 2A). It is evident that the Watchman system and the PLAATO system are similar in terms of material, designing concept (occlusive), and delivery. In the pilot Watchman study, a total of 75 patients were recruited but only 66 patients successfully received the implants. Nine patients did not receive the device due to anatomical difficulty or device wire malfunction. Due to complications (5 in the first 16 cases) the device and delivery system were modified to the current format. Pericardial effusions occurred in 2 of the 75 cases (2.6%). At 45 days TEE follow-up, 93% of devices showed successful sealing of LAA according to protocol. Overall, the preliminary data suggested LAAO with the first-generation Watchman system was safe and feasible [61].

In the randomized non-inferiority PROTECT AF trial comparing Watchman to warfarin [62], total of 707 eligible patients were randomly assigned in a 2:1 ratio to Watchman 2.5 implantation or warfarin with target international normalized ratio (INR) 2–3. Primary composite endpoint of stroke, cardiovascular death, and systemic embolism (SE) was analyzed by intention to treat. Follow-up of 1065 patient-years demonstrated that the primary efficacy event rate occurred at 3.0 per 100 patient-years in the intervention, 4.9 per 100 patient-years in the control, with the probability of non-inferiority of the intervention being more than 99.9%. This trial offered strong evidence favoring the efficacy of percutaneous closure of the LAA with Watchman 2.5 and thereby provided an alternative strategy to chronic oral anticoagulant therapy for stroke prophylaxis in patients with NVAF. However, two major concerns were raised regarding PROTECT AF. The first was inclusion of NVAF with a relatively low CHADS2 score (2.6 for each group), and the second was periprocedural complications which were mainly driven by pericardial effusion requiring intervention. Another concern for the PROTECT AF was a higher dropout rate for the Warfarin group with extended follow-up of 3.8 years because of the patients’ desire for NOAC/DOAC and perceived lack of benefit from continuing warfarin (i.e., bleeding complications). However, analyzed by the time in therapeutic range prior to withdrawal, the higher-risk warfarin patients withdrawing biased the study against the device group [63]*.* Despite significant improvement in procedural safety and clinical benefit by combined analysis of PROTECT AF trial and Continued Access Protocol (CAP) Registry [64, 65] with Watchman 2.5 device, some of these concerns remained. Consequently, the prospective randomized PREVAIL Trial was designed and conducted with data published in 2014 [66]. A total of 407 NVAF patients were enrolled in a 2:1 design for Watchman 2.5 (Mean CHADS-Vasc 3.8) and warfarin (Mean CHADS-Vasc 3.9) for a mean follow-up of 18 months. Two efficacy and one safety co-primary endpoints were assessed. LAAO with Watchman 2.5 was found noninferior to warfarin for ischemic stroke prevention or systemic embolism (SE) >7 days post-procedure. Adverse events were low and numerically comparable in both arms. This trial confirmed that as operators gained experience the periprocedural complications were significantly improved [64, 65], and provided additional data that LAAO with Watchman 2.5 is a reasonable alternative to warfarin therapy for stroke prevention in patients with NVAF. A critical issue about this study is the failure to meet the noninferiority of the pre-specified first co-primary end point (composite of stroke, systemic embolism, and cardiovascular/unexplained death) by 18-month follow-up although the events in both groups were similar. Part of the reason was the extremely low stroke/TIA rate (0.71 per 100 patient-years) in warfarin group with a CHADS2 score of 2.6. Other large randomized controlled trials of stroke prevention using NOAC/DOAC in NVAF that had included a warfarin demonstrated a much higher event rate between 1.6–2.2 per 100 patient-year [66] in the warfarin group. A significantly low incidence rate in the warfarin group and a relatively small number of patients enrolled might have blunted the ability to detect a noninferiority for PREVAIL.

In Europe the EWOLUTION study [67] was designed to collect prospective data on Watchman 2.5 performance in a real-world clinical setting in a high-risk patient cohort. A total of 1025 subjects mean-aged 73.4 were scheduled for implant in the study in 47 centers in 13 countries. The study population was deemed high risk, having a mean CHADS-Vasc of 4.5 and HAS-BLED score of 2.3 (73.3% contraindicated for oral anticoagulation). Findings revealed a high success in device implantation (98.4%) and efficacy in ischemic stroke prevention. The major bleeding rate was 2.6%, although predominantly (2.3%) non-procedure-device related.

Watchman 2.5 became the first LAAO device approved in the US (March 2015) although it was removed from the US market shortly after the second-generation device, Watchman FLX was released in August 2020. The 5-year outcomes of the PREVAIL and PROTECT AF clearly demonstrated that device provided stroke prevention comparable to warfarin, with additional reductions in major bleeding, hemorrhagic stroke, cardiovascular and all-cause mortality [68, 69].

In 2014, even before the US FDA approval of the Watchman 2.5 device, the National Cardiovascular Data Registry (NCDR) considered developing an LAAO Registry. NCDR, the Society of Cardiovascular Angiography and Interventions (SCAI), US FDA, Centers for Medicare and Medicaid Services (CMS), and Boston Scientific were all participants, collecting data in 38,158 Watchman procedures performed by 1318 physicians in 495 hospitals in the United States from January 2016 to December 2018. Description of this “real world” experience [70] revealed a major in-hospital adverse events of 2.16% including pericardial effusion requiring intervention (1.39%) and major bleeding (1.25%), while stroke (0.17%) and death (0.19%) were rare. Of note, the real-world patients were older (mean 76.1 years) and had a higher mean CHADS-Vasc score (4.6) and HAS-BLED score (3.0) compared to previous Trial and Registry patients. The median number of LAAO procedures performed annually for hospitals was 28 and for physicians was 12. A separate meta-analysis included 19 randomized controlled trials with a total of 87,831 patients with NVAF receiving anticoagulants, anti-platelet therapy (APT), placebo or LAAO [71]. Analysis using warfarin as the common comparator demonstrated efficacy benefit favoring LAAO as compared with placebo and APT, and similarity to NOACs/DOACs for preventing mortality and stroke or systolic embolism, with similar bleeding risk. While these studies have limitations in terms of design and patient selection, they do provide reassuring evidence.

### 3.4 Watchman FLX: The Next Generation Device

Although Watchman 2.5 was associated with a relatively low procedure-related complications with increasing clinical experiences, limitations of this device including the size, re-capturability, perforation, peri-device leak, and device-related thrombus (DRT) persisted in clinical practice. To address these concerns the second-generation device, Watchman FLX (Fig. 2B) was designed and has been available in Europe since November 2015. Major modifications in the second-generation device included (1) size, (2) shape, and (3) fixation anchor (Fig. 2C). The new features of Watchman FLX allow not only a wide range of compression (10–30% vs 8–20% recommended for Watchman 2.5) but also full recapture and redeployment repeatedly before final device release.

PINNACLE FLX, the clinical trial that led to the US approval of Watchman FLX, enrolled 400 patients in 2018; the mean-age was 73.8 with a mean CHA2DS2-Vasc score of 4.2 and a HAS-BLED score of 2.0. The new device was found to have very low incidence of pericardial effusion requiring intervention (0.5%, 4/400) during follow-up of 7 to 340 days post implantation. Procedural success was 100% at implant with 0% peri-device leak by 12-month TEE [72]. The clinical impact of Watchman FLX was further ascertained by comparing in-hospital outcomes for the Watchman FLX with Watchman 2.5. Using data from NCDR the primary endpoint of in-hospital major adverse events (MAE) was compared between Watchman FLX and Watchman 2.5 with each arm included 27,013 patients [73]. MAE was significantly lower in the Watchman FLX group (1.35% vs 2.40%). In addition, the in-hospital mortality (0.12% vs 0.24%), major bleeding (1.08% vs 2.05%), cardiac arrest (0.13% vs 0.24%), and device embolization (0.02% vs 0.06%) were also significantly lower while myocardial infarction, stroke, and major vascular complications did not differ between groups. Watchman FLX currently dominates the US market, while both Amulet and Watchman FLX share most of the European market.

### 3.5 LAmbre™

In Europe LifetechScientific (Shenzhen, China) received CE-Mark approval for the LAmbre closure system in June 2016. The device is self-expanding and constructed from a nitinol mesh and polyester membranes. It consists of a hook-embedded umbrella (lobe) and a cover (disc) connected by a short central waist which functions as an articulating compliant connection between the cover and the umbrella, allowing the cover to self-orient to the cardiac wall. Two different types of devices were designed to accommodate single- and double-lobe LAA anatomies, with single-lobe sizing between 16 to 36 mm and double-lobe sizing between 16 to 26 mm.

Preclinical data in animal experiments showing the feasibility with high success rate for the “an umbrella in the left atrial appendage” were published in 2013 [74, 75]. Preliminary study in 15 patients [76] and an initial European experience in 60 patients [77] demonstrated an excellent implant success rate, favorable implant properties, and very low incidence of complications with good mid-term performance regarding stroke prevention. A prospective, multicenter study [78] conducted in 153 NVAF patients with CHADS2 score ≥1 demonstrated high success (152/153) and relatively low complication rate (5/153). A systematic review including 403 NVAF patients [79] demonstrated excellent implantation success rate, promising follow-up clinical data, and favorable properties for also challenging LAA anatomies. First-in-Human implantation of the LAmbre device in the United States was described in 2021 [101] and the clinical trial is ongoing. Wide clinical application will have to await US FDA approval. In any event, limited clinical comparison studies appear to suggest that LAmbre Amulet and Watchman 2.5 all exhibit high implant success rates, low risk of periprocedural adverse events, and good clinical outcomes [76, 102, 103, 104].

### 3.6 WaveCrest™

The WaveCrest (Biosense Webster, Diamond Bar, CA, USA) is a single lobe LAAO device. Initial preclinical testing and first-in-man studies were performed in New Zealand in 2010. Enrolment in the WaveCrest 1 phase II clinical study began in 2011 and acute results in 63 patients were presented at EuroPCR 2013 [80]. The current generation device (WaveCrest 1.3) comes in three sizes (22, 27, and 32 mm) to cover LAA ostia between 18 and 30 mm. The WaveCrest 1.2 device received CE-Mark approval in Europe in 2013. In the more recent WaveCrest 1.3 device, the frame perimeter is provided with 20 fixation hooks to anchor the device to the LAA and enhance stability. The major differences between the WaveCrest 1.2 and 1.3 devices are that the 1.3 device has more anchors and an extended ePTFE cover. Although this device has been granted a CE-Mark since 2013 and marketed in Europe, it is not yet approved in the US.

A pivotal trial within the United States, WAVECREST II, a prospective, multicenter, randomized, active controlled clinical trial was designed to evaluate the safety and effectiveness of this LAAO System. Subjects (n = 1550) were to be randomized in a 1:1 ratio to the treatment arm (WaveCrest II) or the control arm (Watchman 2.5), with the hypothesis that safety and effectiveness of the WaveCrest II device are non-inferior to the comparator Watchman 2.5. The trial enrolled the first patient in January 2018 [81] and is still “active” but not recruiting as the Watchman 2.5 device has been removed from the US market since March 2021. Going forward any device-device comparison will have to be performed using Watchman FLX or Amulet as the control arm.

### 3.7 LARIAT

Technically, LARIAT is not a “device” but rather a loop suture delivering system that is designed to ligate the appendage at the base/ostia. LARIAT system has been described in detail in preclinical studies [82, 83] as well as in human application as an accompanying procedure during mitral valve surgery or AF ablation more than a decade ago [84]. LARIAT uses a snare to deliver a suture loop ligating the LAA at the base from the epicardial surface, and thereby exclude it from the left atrium [83, 84, 85, 86].

The LARIAT technique requires two accesses: endocardial transseptal puncture for balloon catheter and magnet wire placements and epicardial loop suture and magnet wire delivery. At the beginning of the procedure a 12 F catheter is placed in the pericardial space to deliver an adjustable, pre-tied suture loop around the LAA. The new system ${\mathrm{LARIAT}}^{+}$ has a larger snare accommodating LAA diameters up to 45 mm. Then an 8 F catheter with a radiopaque inflatable (up to 20 mm) balloon tip is placed in the LAA via a standard transseptal sheath (8.5 F) to aid in precise location of the epicardial suture loop. The first endocardial magnet-tipped guidewire is placed near what the operator perceives to be the apex of the LAA. Then a second endocardial magnet-tipped guidewire is placed at the tip of the LAA to establish a ‘stable connection’ between the wires. Initial clinical experience demonstrated that LAA closure with the LARIAT device could be performed effectively in 85/89 patients. Complete ligation by TEE was 95% at 3 months and 98% at 12 months, with acceptably low access complications and periprocedural adverse events [85]. Initially, patients required at least overnight or longer hospital stay, with a pericardial drain left in place for overnight or longer [87, 88, 89].

Pericardial access has long been and remains challenging for most electrophysiologists and interventional cardiologists. A multicenter registry of 712 consecutive patients undergoing LAA ligation with LARIAT at 18 US hospitals [90] demonstrated successful deployment in 682 patients (95.5%) and complete closure in 669 patients (98%). Nonetheless, acute perforation of 3.5%, delayed pericardial and pleural effusion of 4.78% after discharge, follow-up TEE showing a leak of 6.5%, and a thrombus in 2.5% of the patients were significant. Despite a favorable collective European experience in 141 patients demonstrating the feasibility of LAA exclusion using ${\mathrm{LARIAT}}^{+}$ with 97.1% complete closure by TEE at 6 months [91], an American study of 306 patients [92] reported a much higher post procedural leak of 26.5% at one month and 19.6% at 6 months of TEE follow-up. At a median follow-up period of 15.9 months, 9 patients developed thromboembolic events (2.9%). It is reasonable to assume that before randomized, controlled, prospective trials against newer anticoagulants or Watchman FLX/Amulet with long term efficacy and safety data are available clinical applications of LARIAT system will be limited.

### 3.8 Ultraseal

The Ultraseal device (Cardia, Eagan, Minnesota) is a self-expandable bulb-and-sail nitinol occluder which received Conformité Européenne (CE-Mark) approval in March 2016. The device is composed of 2 parts: a soft distal bulb and a distal polyester layer. The delivery system is 10 F to 12 F. The fully retrievable device allows it to be positioned and re-positioned as needed to ensure accurate placement.

Initial experience with the Ultraseal I device demonstrated safety and feasibility in 12 NVAF patients: At 45-day follow-up there was no bleeding, stroke, pericardial effusion, or device embolization in this small study group [93]. Residual leak >5 mm was not observed by TEE in any case. DRT was found in one patient, without clinical consequences. Another study in 23 consecutive NVAF patients also demonstrated high success rate of implantation (21/23) and extremely low complication rate at a mean follow-up of 166 ± 80 days [94]. In multicenter experience of 126 patients from 15 Canadian and European centers [95] the device was successfully implanted in 97% of patients, with major periprocedural adverse events (pericardial effusion, stroke, device embolization) occurring in only 3 (2.4%) instances. At a median follow-up of 6 months the rates of stroke and transient ischemic attack were 0.8% and 0.8%, respectively, with no systemic emboli. Despite low periprocedural complications reported by previous studies, 2 out of 18 patients were found to have device fractures in another case series [96].

Recently in a multicenter international registry [97] comprising 52 NVAF patients with 6-month follow-up the modified Ultraseal II seems to have reaffirmed the high success implantation rates, low incidence of peri-procedural complications, and improved device safety profile. Larger studies with longer clinical follow-up, especially incorporating comparison with the existing US FDA approved two devices (Watchman FLX and Amulet) are needed to further evaluate safety and efficacy before recommending this device for wide clinical application.

### 3.9 Conformal Left Atrial Appendage Seal

The Conformal Left Atrial Appendage Seal (CLAAS) device (Conformal Medical, Inc., Nashua, NH, USA) includes an implant and a delivery system (sheath and delivering catheter). The implant (27 mm and 35 mm diameter options) is made of a self-expandable cylindrical nitinol endoskeleton covered by porous polyurethane-carbonate matrix foam. The distal portion of the form cup (LAA side) extends beyond the endoskeleton to serve as an atraumatic leading edge during device implantation. There are two rows of anchors: 10 each for the 27 mm device and 12 each for the 35 mm device. The foam is highly conformable and has a porous surface area promoting tissue ingrowth from the LAA. The 27 mm device fits an 18 F short venous access sheath, and the 35 mm device fits a 20 F sheath. The implant is attached to the delivery catheter with a flexible suture tether for recapture and redeployment before final device release. Preclinical assessment performed in 7 dogs demonstrated the conformability of the CLAAS implant and its ability to seal the LAA [98]. First clinical experience reported that the device could be implanted in 18 of 22 NVAF patients with a CHA2DS2-Vasc score of ≥4 and HAS-BLED score of ≥3 [99]. TEE at 45 days found one leak >5 mm due to unappreciated large posterior LAA lobe at the time of implantation, and one device-related thrombosis which resolved with prolonged anticoagulation. Four patients failed to receive the device due to the unavailability of the large 35 mm device at the time of implantation (the 27 mm device was tried but recaptured and retrieved due to the inadequate seal). There were no periprocedural strokes, pericardial effusions requiring intervention, or systemic or device embolization. This first-in-human study as part of the ongoing device feasibility trial (NCT03616028) appears to show the clinical feasibility of the CLAAS device for LAAO. Another study in 15 NVAF patients with a CHADS-Vasc score of 4.1 and a lower HAS-Bled score (1.4) demonstrated 100% success in device implantation with no procedure/device-related complications requiring intervention [100]. Adequate LAA seal in all patients was confirmed by follow-up TEE up to 12 months post-implant, with one device-related thrombus detected at 6 months. This latter study was performed using intracardiac echocardiography guidance. In brief, although experience to date is small, LAAO with the CLAAS device guided by intracardiac echocardiography (ICE) imaging appears to be feasible with encouraging 1-year clinical outcomes. Nevertheless, it has yet to receive CE-Mark approval and a larger randomized, controlled trial (The CONFORM Pivotal Trial) comparing CLAAS with Watchman FLX and Amulet is ongoing in the US currently (Table 2).

**Table 2.** **Ongoing Clinical Trials. Dates denote actual study starting date and estimated primary completion date**.

Name	NCT #	Subjects	Dates
OPTION N = 1600	NCT03795298	Comparison of Anticoagulation with Left Atrial Appendage Closure after Atrial Fibrillation Ablation	05/2019–11/2024
CHAMPION-AF N = 3000	NCT04394546	WATCHMAN™ FLX Versus NOAC for Embolic ProtectION in in the Management of Patients with Non-Valvular Atrial Fibrillation	10/2020–12/2027
CATALYST N = 2650	NCT04226547	Clinical Trial of Atrial Fibrillation Patients Comparing Left Atrial Appendage Occlusion Therapy to Non-Vitamin K Antagonist Oral Anticoagulants	07/2020–12/2024
CLOSURE-AF N = 1512	NCT03463317	Left Atrial Appendage CLOSURE in Patients With Atrial Fibrillation Compared to Medical Therapy	02/2018–09/2023
OCCLUSION-AF N = 750	NCT03642509	Left Atrial Appendage Occlusion Versus Novel Oral Anticoagulation for Stroke Prevention in Atrial Fibrillation	01/2019–01/2024
STROKECLOSE N = 750	NCT02830152	Prevention of Stroke by Left Atrial Appendage Closure in Atrial Fibrillation Patients After Intracerebral Hemorrhage: A Multicenter Randomized Clinical Trial	05/2017–12/2027
ASAP-TOO N = 481	NCT02928497	Assessment of the WATCHMAN™ Device in Patients Unsuitable for Oral Anticoagulation	02/2017–12/2025
ASPIRIN LAAO N = 1120	NCT03821883	Aspirin Discontinuation After Left Atrial Appendage Occlusion in Atrial Fibrillation	06/2020–06/2022
CLEARANCE N = 550	NCT04298723	Comparison of LAA-Closure vs Oral Anticoagulation in Patients With NVAF and Status Post Intracranial Bleeding	06/2020–06/2025
The CONFORM Pivotal Trial N = 1600	NCT05147792	An Evaluation of the Safety and Effectiveness of the Conformal CLAAS System for Left Atrial Appendage Occlusion	05/2022–08/2026

Abbreviations: NCT, national clinical trial; NOAC, novel oral anticoagulant; LAA, left atrial appendage; NVAF, non-valvular atrial fibrillation; CLAAS, Conformal left atrial appendage seal.

## 4. LAAO: Current Clinical Status and Ongoing Clinical Trials

### 4.1 US FDA Approved Devices

Currently, both Watchman FLX and Amulet are US FDA approved and being used in the US, with the former dominating the marketplace. Both devices share a major part of European market with other CE-Mark approved devices also being in use or in clinical trials. The detailed market shares of various LAAO devices in China and other Asian countries are yet unclear*. *Randomized, controlled trials comparing the clinical performance of the two devices are lacking currently. With one year follow-up in a cohort of 51 patients (25 Watchman 2.5, 26 Amulet) the peri-device leak was found significantly higher in the Watchman 2.5 group [105]. A single center experience comparing Amulet (n = 150) and Watchman FLX (n = 150) demonstrated a significantly lower peri-device leak in the latter group [106]. A meta-analysis including 25 studies of 4186 patients (Amulet = 3187; Watchman FLX = 999) seems to suggest that Watchman FLX is associated with a lower incidence of periprocedural adverse events including peri-device leak [107].

### 4.2 Special Clinical Situations 

Four clinical situations that are commonly encountered in current LAAO therapy merit consideration: advanced age, impaired kidney function, LAAO at the time of NVAF ablation, and LAAO in high stroke risk and high bleeding risk NVAF patients on NOAC/DOAC. Patient’s age does not seem to be a factor in recommending LAAO therapy based upon available data. A recent analysis of 36,065 LAAO recipients using Watchman device, of which 34.6% were aged 80 years or older, provides support in this regard [108]. After adjusting for potential confounding variables, advanced age was not associated with procedure-related adverse outcomes including major complications, prolonged length of hospital stays, or increased hospitalization costs. On the other hand, inpatient mortality was increased probably reflecting a frail population with higher co-morbidities including congestive heart failure, renal failure, and peripheral vascular disease in the elderly. Analysis of EWOLUTION Registry demonstrated that the procedural success was high and similar (98.8% vs 98.5%) and there were no differences in 7-day device- or procedure-related adverse event rates for those aged 85 year older or younger [109]. Another multicenter registry study of 1053 subjects using ACP I also demonstrated that LAAO was associated with similar procedural success (97.3%) in patients aged <75 and ≥75 years, with stroke and major bleeding rates being similar at a mean follow-up of 16.8 months [110]. Patient’s renal function status also does not seem to affect LAAO therapy. It is well-known that patients with chronic kidney disease and especially end-stage renal disease are at increased complications due to bleeding on oral anticoagulation. NOACs/DOACs may be preferrable to warfarin [111] in NVAF patients with impaired renal function. Available evidence has shown that in those patients LAAO therapy is safe and effective and can be considered as an alternative to NOACs/DOACs for stroke prevention [111, 112, 113].

It is reasonable to consider undertaking AF catheter ablation and LAAO at the same time because the two percutaneous interventions share some procedural issues and technical requirements. In clinical terms the combined procedure could be deemed equivalent to combining antiarrhythmic drugs for AF symptomatic improvement and anticoagulation for stroke prevention. The earliest report in 30 patients published a decade ago demonstrated the safety and feasibility [114], and this was further supported by pooled data analysis [115]. Propensity score matched analysis from the US National Readmission Database demonstrated an annual growth rate of 63% between 2016 to 2019, with no significant difference in major adverse cardiovascular events (MACE) and all-cause 30-day readmission rates among combined procedure patients compared with matched LAAO-only or catheter ablation-only patient [116]. A retrospective analysis of 1114 patients who underwent the combined procedure in China supported the safety and long-term efficacy [117]. Model analysis suggested that in symptomatic NVAF patients with high stroke and bleeding risk who are planned for catheter ablation, the combined procedure may be a cost-effective therapeutic option and more beneficial to those with CHADS-VASc risk score ≥3 [118]. Randomized controlled data will have to await the outcome of the OPTION trial (Table 2).

Current clinical guidelines [6, 7] regarding LAAO therapy were written at a time when neither Watchman FLX nor Amulet had been approved. The IIb recommendation in both the ACC/AHA/HRS and the ESC guidelines stated that percutaneous LAA occlusion may be considered in patients with AF at increased risk of stroke who have contraindications to long-term anticoagulation. Given subsequent increased clinical experience, both improved device technology and periprocedural complication rates, and favorable long-term efficacy and safety outcomes in large number of patients, it is likely that the next guidelines will offer elevation in recommendation, at least in certain populations of NVAF patients. To this point, the PRAGUE-17 trial [119, 120] has demonstrated the non-inferiority of LAAO in composite end points including cardiovascular death, all stroke/TIA, and clinically significant bleeding events at both mid-term follow-up of 19.9 months and long-term follow-up of 3.5 years in a high stroke risk/high bleeding risk population (CHA2DS2-Vasc 4.7 ± 1.5; HAS-BLED 3.1 ± 0.9) on NOAC/DOAC. Non-procedural bleeding is significantly reduced at long-term follow-up. Outcome data comparing LAAO with NOAC/DOAC in average risks of stroke and bleeding NVAF population also awaits ongoing clinical trials including CHAMPION-AF and CATALYST (Table 2).

### 4.3 Imaging Techniques

The imaging techniques for LAAO have also been evolving. TEE with 2D and color doppler and fluoroscopy were the original imaging techniques for guiding LAAO and were required for all pivotal clinical trials. These techniques are currently the major and likely remain to be the dominant modalities for LAAO therapy in the future. Over the past 20 years other imaging techniques such as computed tomography (CT), ICE, and micro-transesophageal echocardiography (micro-TEE) are also being evaluated and adopted to (for) the application of LAAO. Detailed discussion for each of these techniques is beyond the scope of this review but TEE with both 2D and 3D imaging [121, 122] and high-resolution CT [123] are the most frequently used techniques for preprocedural assessment of LAA anatomy, ruling out intracardiac thrombus, and post procedural follow-up regarding device seal of the appendage and device related thrombosis. TEE is also the main intraprocedural imaging technique guiding the device implantation. The undesirable features of TEE include its invasiveness, requirement for fasting, and general anesthesiology support during device implantation. In addition, some patients may have pre-existing esophageal pathologies such as esophageal stricture or vein varices that make the probe placement difficult and risky, especially in patients with significant coagulopathy. The non-invasiveness of CT with remarkably high spatial resolution makes it ideal for pre- and post-procedural LAAO evaluations and has been used with increasing frequency. The requirement for contrast injection, especially in those with significant kidney disease, and radiation exposure are the main limitations. ICE [124] is used primarily for guiding transseptal puncture and device deployment. The technique is familiar to most electrophysiologists. Major advantages of ICE include avoidance of TEE probe placement and general anesthesiology support during the procedure. Limited catheter maneuverability and imaging quality, requirement for dilation of transseptal or additional transseptal puncture to advance the catheter into the left atrium, and additional venous access site are the main disadvantages. ICE with 3D and 4D capabilities may improve the imaging quality. A recent meta-analysis seems to suggest that TEE and ICE guided LAAO procedures have equivalent clinical outcomes, including procedural success, fluoroscopy time, total procedural time, and complication rate [125]. The miniaturized multiplane micro-TEE probe (Philips Medical Systems, Andover, MA, USA) was originally designed for infants. The transducer tip width and height are only 7.5 mm and 5.5 mm, respectively. In a study [126] performed under conscious sedation micro-TEE guided LAAO was found to be safe and effective compared to the traditional TEE guided procedures which otherwise require deep sedation by anesthesiologist. With multiple imaging modalities available selection of LAAO related imaging techniques could be individualized, based on patient’s clinical comorbidity, implanter proficiency, and institutional support.

### 4.4 Post LAAO Medical Therapy

Currently there are no randomized controlled trials comparing post LAAO anticoagulation and antiplatelet regimen in terms of medications and duration, and therefore the optimal post-LAAO medical therapy remains to be defined. In PROTECT AF and PREVAIL warfarin for 45 days was recommended post-Watchman 2.5 implantation. Thereafter clopidogrel replaced warfarin for another 4.5 months. ASA was recommended indefinitely post implantation. Coincidental with USFDA approval of Watchman 2.5 in March 2015 there has been increasing use of NOAC/DOAC in NVAF patients. In those patients NOAC/DOAC could replace warfarin for 45 days post implantation. The same recommendation applies to Watchman FLX after its approval in August 2020, although due-antiplatelet (Clopidogrel plus ASA) regimen post Watchman FLX implantation was also approved by USFDA in 2022. Six months after successful device implantation patients may stop taking clopidogrel but continue ASA indefinitely. Post Amulet implantation will be due-antiplatelet for 6 months and thereafter ASA indefinitely based on the Amulet IDE trial [58]. In neither the US nor the EU current cardiology practice routinely follows the post-procedure treatment protocols studied in pivotal trials, with various antithrombotic and anticoagulation regimens being reported [127, 128]. In the absence of randomized controlled clinical trials post LAAO medical regimens need to be individualized taking into consideration of available trial data and device vendor recommendations, individual patient’s stroke and bleeding risk profiles as well as other comorbidity such as hypercoagulable state or kidney function, characteristics of different device types, implantation outcome including residual peri-device leak, depth in the LAA, and procedural complications, and post implantation (45–90 days) TEE/CT imaging information (residual leak or DRT). If significant peri-device leak or DRT is present, anticoagulation should be prolonged until DRT and leak are resolved, or leak becomes acceptable.

At the present time it might be fair to argue that for NVAF patients who are at substantial risk for stroke, yet in whom pharmacologic anticoagulation presents excessive bleeding risk or who have exhibited poor drug compliance, any CE-Mark approved LAAO device can be selected. However, many LAAO therapy-specific and device-specific questions remain to be addressed in ongoing clinical trials (Table 2).

## 5. Future Perspectives

While considerable progress has been made in trans-catheter LAAO therapy, there are still many questions to be addressed. Some of the more important include:

### 5.1 Clinical Concerns

(1) Whether LAAO would be more efficacious and safer compared to DOACs/NOACs, in those NVAF patients who do not have high bleeding risk but still need stroke prevention is uncertain?

(2) Would LAAO or antiplatelet agents be the preferred treatment option for NVAF patients who have contraindications to anticoagulation?

(3) What is the optimal post-LAAO regimen? Current therapies range from short-term anticoagulation using warfarin or DOAC/NOAC to single or double antiplatelet agents or no therapy at all.

(4) Is there a difference between the two currently available US FDA approved devices, (i.e., Watchman FLX and Amulet) regarding procedural safety and long-term efficacy?

(5) Currently high-quality long-term follow-up data are lacking for those LAAO devices that are CE-Mark approved but not-yet US FDA-approved devices. Should head-to-head clinical trials versus Watchman FLX or Amulet be required?

(6) What is/are the best/most appropriate preprocedural, intraprocedural, and follow-up imaging modalities: TEE, Micro-TEE, coronary computed tomography angiography (CCTA), or ICE?

(7) Would LAAO be a replacement or just complimentary therapy for recurrent stroke/TIA patients who are already on appropriate anticoagulation?

(8) What are the most appropriate treatment options for patients who have had optimal LAAO and appropriate post implantation antiplatelet/anticoagulation therapy, yet still developed stroke or TIA?

### 5.2 Industry Issues

For the medical technology industry, future device design and modification might focus on:

(1) Minimizing risks of device-related thrombosis and periprocedural pericardial effusion/tamponade,

(2) Improving ease of device delivery, stability, and retrieval use,

(3) Designing smaller French size delivery systems to minimize groin access complications, and

(4) Providing flexible/steerable sheath mechanisms to facilitate device release for various LAA anatomies.

### 5.3 Academic Concerns

The academic community also has a significant role to play in contributing to advances of LAAO therapy as well:

(1) Which type/s of LAA anatomy would possess the highest risk for thrombus formation/stroke/TIA and therefore benefit the most from LAAO?

(2) What are the hemodynamic changes, mechanical, and electrical remodeling/reverse remodeling after LAAO [119, 120]?

(3) Are there significant biochemical and/or endocrinologic effects after LAAO and will those changes affect clinical outcome [129, 130]?

(4) Is LAAO pro-arrhythmic, anti-arrhythmic, or arrhythmia-neutral?

(5) Is device intervention cost-effective? Does LAAO therapy remain cost-effective in the elderly where operative risk may be greater and duration of anticoagulant therapy being relatively short?

### 5.4 General Topics

For clinicians, industry, and academic communities, what is the role of LAAO for valvular AFs who are currently excluded from LAAO trials? The world-wide burden of valvular AF is substantial with about 30% of AF patients having some form of valvular heart disease detectable by echocardiography [131]. Further, in less-well developed countries the prevalence of rheumatic heart disease remains high, and most cases of AF are attributable to rheumatic heart disease and would be considered valvular AF [132]. Answers to these questions will undoubtedly impact the ultimate utility of LAAO therapy.

## 6. Conclusions

Trans-catheter LAAO therapy has achieved a level of clinical acceptability in terms of embolism protection and procedural safety. Further, a number of innovative devices are currently either approved for use in the USA or in Europe, or both. Other LAAO devices and strategies are currently undergoing clinical evaluation; as more become clinically available, the options available for various anatomic and clinical circumstances will grow. The next step will then be updating LAAO clinical guidelines to keep pace with both technological advances, and the inevitable improved understanding of the appropriate LAAO clinical landscape.

## Abbreviations

ACC, American college of cardiology; ACP, Amplatzer cardiac plug; ACP II, Amulet; AF, atrial fibrillation; AHA, American heart association; APT, antiplatelet; CAP, continued access protocol; CCTA, coronary computed tomography angiography; CE-Mark, Conformité Européenne (Commercial Sale of Licensed Product in the EU); CMS, centers for medicare and medicaid services; CT, computed tomography; DOACS, direct oral anticoagulants; DRT, device related thrombosis; ePTFE, expanded polytetrafluoroethylene; ESC, European society of cardiology; HRS, heart rhythm society; ICE, intracardiac echocardiography; IDE, investigational device exemption; INR, international normalized ratio; LA, left atrium; LAA, left atrial appendage; LAAO, left atrial appendage occlusion; MACE, major adverse cardiovascular events; MAE, major adverse events; Micro-TEE, micro-transesophageal echocardiography; NCDR, national cardiovascular data registry; NOACS, novel oral anticoagulants; NVAF, nonvalvular atrial fibrillation; OAC, oral anticoagulation; PLAATO, percutaneous LAA transcatheter occlusion; SCAI, society of cardiovascular angiography and interventions; SE, systemic embolism; TEE, transesophageal echocardiography; TIA, transient ischemic attack; US FDA, United States food and drug administration.
